# Weed Suppressing Potential and Isolation of Potent Plant Growth Inhibitors from *Castanea crenata* Sieb. et Zucc

**DOI:** 10.3390/molecules23020345

**Published:** 2018-02-07

**Authors:** Phung Thi Tuyen, Tran Dang Xuan, Truong Thi Tu Anh, Truong Mai Van, Ateeque Ahmad, Abdelnaser Abdelghany Elzaawely, Tran Dang Khanh

**Affiliations:** 1 Faculty of Forest Resources and Environmental Management, Vietnam National University of Forestry, Xuan Mai, Hanoi 156200, Vietnam; phungtuyen@gmail.com; 2 Graduate School for International Development and Cooperation, Hiroshima University, Higashi Hiroshima, Hiroshima 739-829, Japan; tuanhbp@gmail.com (T.T.T.A.); truongmaivan1991@gmail.com (T.M.V.); 3 Process Chemistry and Technology Department, CSIR-Central Institute of Medicinal and Aromatic Plants, P.O.-CIMAP, Lucknow-226015, India; ateeque97@gmail.com; 4 Department of Agricultural Botany, Faculty of Agriculture, Tanta University, Tanta 31527, Egypt; elzaawely@agr.tanta.edu.eg; 5 Department of Genetic Engineering, Agricultural Genetics Institute, Pham Van Dong Street, Hanoi 122300, Vietnam; khanhkonkuk@gmail.com

**Keywords:** *Castanea crenata*, allelochemicals, 2α,3β,7β,23-tetrahydroxyurs-12-ene-28-oic acid, inhibitory potential, *E. crus-galli*, monocot weeds, dicot weeds

## Abstract

This study isolated, determined, and quantified plant growth inhibitors in Japanese chestnut (*Castanea crenata* Sieb. et Zucc), a deciduous species native to Japan and Korea. In laboratory assays, *C. crenata* leaves showed strong inhibition on germination and seedling growth of *Echinochloa crus-galli* (barnyardgrass), *Lactuca sativa* (lettuce), and *Raphanus sativus* (radish). Laboratory and greenhouse trials showed that leaves of *C. crenata* appeared as a promising material to manage weeds, especially the dicot weeds. By GC-MS and HPLC analyses, gallic, protocatechuic, *p*-hydroxybenzoic, caffeic, ferulic, ellagic, and cinnamic acids were identified and quantified, of which ellagic acid was present in the highest quantity (2.36 mg/g dried leaves). By column chromatography and spectral data (^1^H- and ^13^C-NMR, IR, and LC-MS) analysis, a compound identified as 2α,3β,7β,23-tetrahydroxyurs-12-ene-28-oic acid (**1**) was purified from the methanolic leaf extract of *C. crenata* (0.93 mg/g dried leaves). This constituent showed potent inhibition on growth of *E. crus-galli*, a problematic weed in agricultural practice. The inhibition of the compound **1** (IC_50_ = 2.62 and 0.41 mM) was >5 fold greater than that of *p*-hydroxybenzoic acid (IC_50_ = 15.33 and 2.11 mM) on shoot and root growth of *E. crus-galli*, respectively. Results suggest that the isolated the compound **1** has potential to develop natural herbicides to manage *E. crus-galli*. This study is the first to isolate and identify 2α,3β,7β,23-tetrahydroxyurs-12-ene-28-oic acid in a plant and report its plant growth inhibitory potential.

## 1. Introduction

Synthetic agrochemicals have been widely used in agricultural production for weed management because of their excellent economic efficiency and control efficacy [1]. However, the overuse of synthetic chemicals has caused negative impacts on sustainable weed management and the natural environment [2,3]. Therefore, application of natural compounds for weed control in crop production has been widely studied [4,5,6,7,8]. The use of plants with strong allelopathic activity to suppress weed emergence could minimize the use of synthetic herbicides [9]. Many crops, trees, and weeds show strong weed suppressing ability as they possess a wide variety of allelochemicals [10]. Allelochemicals from plants have inhibitory effects on neighboring plants by phytotoxicity and organogenesis induction [11,12]. Allelochemicals belong to different classes of secondary metabolites, such as phenols, benzoic acid and cinnamic acid derivatives, terpenes, alkaloids, carbohydrates, glycosides, and amino acids [1,13,14]. These substances can act through different mechanisms including volatile emission, leaching from leaves, or exudation from roots. Allelochemicals have been reported to have potent inhibition activity on the germination and growth of weeds [5,9,15,16]. The development of bioherbicides derived from allelochemicals is promising for biological control of weeds and pests [5,12,17].

*Castanea crenata* (Japanese chestnut), a tree species native to Japan and Korea, is widely cultivated in several Asian countries [18,19]. *C. crenata* is a deciduous tree, about 17 m tall and 1 m in diameter [20]. Leaf extracts of this species have been reported to display antioxidant, anti-allergic, and anti-amnesic activities, and inhibitory activity against formation of advanced glycation end products (AGEs) [21]. Of these, the anti-AGE activity was the most best when comparing this species with common vegetables (nine species), fruits (14 species), and mushrooms (nine species) in Japan. Proanthocyanidins, oligomeric flavonoids, were identified and quantified in high quantities (380 and 630 mg/g in water and ethanol extracts, respectively) in inner skin, as compared with that of apple and grape [21]. In our previous research, barks, flowers, inner skins, kernels, and leaves of *C. crenata* were evaluated for their antioxidant properties and the chemical components involved in the antioxidant activities of *C. crenata* were investigated*,* including total phenolic, flavonoid, and tannin contents, free and bound-phenolic acids, and flavonoids [22]. In total 13 phenolic acids (gallic acid, protocatechuic acid, catechol, vanilln, sinapic acid, *p*-coumaric acid, benzoic acid, ellagic acid, chlorogenic acid, *p*-hydroxobenzoic acid, syringic acid, ferulic acid, and sinapic acid) and eight flavonoids (isoquercitrin, mycicetin, fisetin, kaempferol, rhamnetin, esculetin, morin, and apigenin) were identified and quantified by HPLC. They were reported to correlate to the antioxidant activities of the plant. Types and concentrations of the identified phenolic acids and flavonoids varied among plant parts of *C. crenata* [22].

The isolation and identification of plant growth inhibitors (allelochemicals) from plants has been extensively investigated in recent years [8]. However, to date, the allelopathy as well as growth inhibitors of *C. crenata* are not yet known. This study aimed to clarify the weed suppression capacity of this chestnut species to examine whether it can be used for weed management, thus leaves of *C. crenata* was used because of their availability. The purification, identification, and quantification of potent plant growth inhibitors from *C. crenata* leaves were also conducted. GC-MS and HPLC were applied to identify and quantify the known phenolic acids by comparing the retention times and mass spectra of the purchased standards, respectively. The analysis of ^1^H- and ^13^C-NMR, IR, and LC-MS spectral data was used to determine compounds other than the phenolic acids and with potent weed management effects, as standards could neither be purchased nor were their spectra data available in the Wiley and NIST libraries.

## 2. Results 

### 2.1. Effects of Leaf Powder on Germination and Root and Shoot Growth of R. sativus, L. sativa, and E. crus-galli 

In general, the leaf powder markedly inhibited germination of *R. sativus, L. sativa*, and *E. crus-galli* at all treatment doses (Table 1). The degrees of inhibition were proportional to the applied doses. The concentration of 0.5 g completely inhibited germination of *L. sativa*. All applied doses of the leaf powder significantly reduced shoot length and root length of the tested plants, except the lowest dose (0.1 g) that slightly promoted shoot height of *L. sativa* and *R. sativus* (*p* < 0.05). At 0.5 g, root growth of all tested plants and seedling growth of *L. sativa* were completely suppressed. It found that the suppression on *L. sativa* > *R. sativus* > *E. crus-galli* (Table 1).

### 2.2. Effects of Aqueous Extracts on Germination and Shoot and Root Growth of L. sativa, R. sativus, and E. crus-galli

Table 2 shows that the effects of the aqueous extracts of *C. crenata* were dose dependent and varied among tested plants. The effects present as the inhibition over control (%). Germination of *S. sativus* was the most inhibited, followed by *L. sativa*, whilst germination of *E. crus-galli* was not affected. In general, the root length was inhibited at stronger levels than the shoot height of the tested plants. At the 1 g/100 mL dose, the root length of *S. sativa*, *S. sativus*, and *E. crus-galli* were inhibited by 46.3, 62.5, and 77.6%, respectively. At the 10–15 g/100 mL doses, the roots of *L. sativa*, *S. sativus*, and *E. crus-galli* were reduced by 87.8–92.7, 98.6–97.2, and 100%, respectively (Table 2). It was suggested that *C. crenata* may possess growth inhibitors to suppress germination and growth of the tested plants. The two plants *L. sativa* and *S. sativus* are commonly used as indicator plants in the allelopathic bioassays because they are more sensitive to plant growth inhibitors at low doses than weeds such as *E. crus-galli* [14]. Thus, findings of this experiment suggested that the inhibition of *C. crenata* on root growth of *E. crus-galli* was selective.

### 2.3. Effects of Ethyl Acetate Extracts on Germination and Growth of R. sativus, L. sativa, and E. crus-galli

Effects of the ethyl acetate extracts of *C. crenata* on the tested plants are presented in Table 3. In comparison with the controls, only root length of *L. sativa* and *E. crus-galli* were affected at 1000 mg/L. At 2000 mg/L, root growth of *R. sativus* and *E. crus-galli* was significantly reduced. At this concentration, root growth of *L. sativa* was completely suppressed. However, effects on germination on the three tested plants were negligible (Table 3).

### 2.4. Elucidation of Chemical Structure of the Compound Isolated from C. crenata Leaf

**Figure 1:**
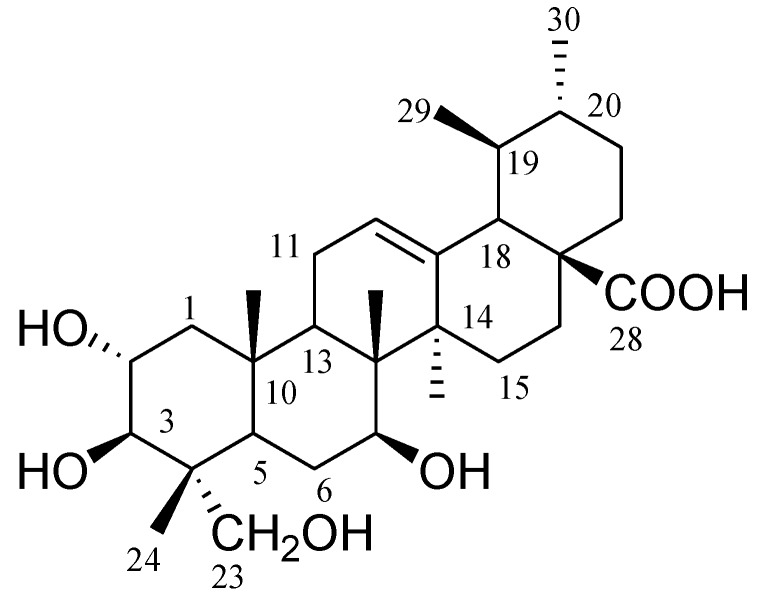
Chemical structure of compound **1** (2α,3β,7β,23-tetrahydroxyurs-12-ene-28-oic acid).

Compound **1** was obtained as a white amorphous powder from the methanol extract. Its molecular formula was deduced as C_30_H_48_O_6_Na, from its HR-ESI MS data that displayed an [M + Na]^+^ ion at *m*/*z* 527.3341 (observed; calculated for C_30_H_48_O_6_Na, *m*/*z* 527.3348) (Supplementary Material, Figure S2). Its IR spectrum showed characteristic absorption bands for hydroxyl groups (3416 cm^−1^), carboxylic group (1706 cm^−1^) and vinylic bond (1648 cm^−1^) (Supplementary Material, Figure S3). The ^1^H- and ^13^C-NMR data of compound **1** (Supplementary Material, Figures S4–S6) was reported in the literature [21]. On the basis of spectroscopic data analysis, the structure of the compound **1** as assigned as 2α,3β,7β,23-tetrahydroxyurs-12-ene-28-oic acid (**1**, Figure 1).

### 2.5. Inhibitory Effects of 2α,3β,7β,23-Tetrahydroxyurs-12-ene-28-oic Acid on Root and Shoot Growth of L. sativa, R. sativus, and E. crus-galli

The inhibitory potential of the isolated compound 2α,3β,7β,23-tetrahydroxyurs-12-ene-28-oic acid was evaluated against *L. sativa*, *R. sativus*, and *E. crus-galli* in comparison with *p*-hydroxybenzoic acid. The inhibition was expressed in IC_50_ value (mM) (Table 4), thus the lower value of the IC_50_ value indicated stronger activity. It was found that the compound 2α,3β,7β,23-tetrahydroxyurs-12-ene-28-oic acid inhibited growth of *E. crus-galli* and *L. sativa* in stronger levels than those of *p*-hydroxybenzoic acid did. The inhibition of 2α,3β,7β,23-tetrahydroxyurs-12-ene-28-oic acid on shoot height and root elongation of *E. crus-galli* were about five-fold stronger than *p*-hydroxybenzoic acid did (Table 4). It is appeared that inhibitory activity of the isolated compound was reflected five-fold greater than that of *p*-hydroxybenzoic acid. The inhibition on root length of *E. crus-galli* even stronger than that of *L. sativa* and *R. sativus*, indicate that the inhibitory potential of the compound **1** was potent to manage emergence of *E. crus-galli*.

### 2.6. Effects of Leaf Powder on Emergence and Growth of Weeds in Natural Soil

#### 2.6.1. Weed Number 

Weed emergence was determined by number of weeds and dry weight. The monocot weeds included *Cyperus microiria* Steud, *Digitaria violascens* Link, and *Fimbristylis littoralis* Gaudich, and the dicot weeds were *Eclipta prostrata* L., *Stellaria media* (L.) Vill., and *Cardamine flexuosa* With.) (Table 5). At the lowest dose (0.10 g), the weed number was either slightly inhibited or promoted, but the levels were not significantly different from the control (Table 5). At concentrations of both 0.25 and 0.5 g, *C. crenata* leaf powder significantly reduced the plant number of both monocot and dicot weeds from 36.7–68%, although the inhibitory level was not markedly different between the two concentrations. At these doses, total number of the weeds were also markedly inhibited (>55%), as compared with the controls.

#### 2.6.2. Dry Weight

*C. crenata* showed selective inhibitory effects on plant number and dry weight of monocot and dicot weeds. At the dose 0.10 g, no significant differences with the control were observed. At the 0.25 g, dry weight of both monocots and dicots were markedly inhibited by 56.4 and 57.7%, respectively. However, at the highest dose of 0.50 g, the dicots were suppressed by 51.0%, but in contrast, the monocots were significantly promoted by 125.6%. The results exhibited that *C. crenata* had selective effects on emergence of monocot and dicot weeds. Growth of the dicots were strongly reduced by 0.25–0.50 g, whilst the inhibition on the monocots (Table 5) was ambiguous and needs further elaboration. 

### 2.7. Effects of Leaf Powder on Emergence and Growth of Weeds in Greenhouse Trials

#### 2.7.1. Weed Number

Three monocot species (*Cyperus microiria, Fimbristylis littoralis,* and *Digitaria violascens*) and three dicot species (*Eclipta alba*, *Stellaria media*, and *Cardamin flexuosa*) were used in the control pots (Table 5). Among the monocot weeds, the effects of *C. crenata* varied among treatments and weed species. The two weeds *C. microiria* and *D. violascens* were promoted, whereas *F. littoralis* was inhibited, although the inhibitory level was not significantly different from the control (Table 6). In contrast, *C. crenata* showed much stronger inhibition on the dicot weeds, except the *C. flexuosa* at the T1 treatment, weed number of the dicots were markedly reduced as compared to the control, of which the inhibitory effect on *C. microiria* was the maximum (Table 6). 

#### 2.7.2. Dry Weight

Similar to weed number, the dry weight of the monocot weeds was strongly promoted in all treatments, whilst the inhibition on the dicot weeds was at much stronger levels, and varied among weed species (Table 6). At all treatments, the dry weight of *E. alba* was significantly inhibited, whereas the inhibition on *S. media* in the T1 treatment was not significantly different from control, and in the T2 and T3 treatments, the dry weight of this weed was strongly promoted. Except for the T1 treatment, emergence of *C. flexuosa* was completely inhibited at T2 and T3 treatments (Table 6). Results of the greenhouse trials were similar to the findings of laboratory experiments that the effects of *C. crenata* on dicot weeds were much stronger on the monocot weeds (Table 5 and Table 6).

### 2.8. Identification and Quantification of Phenolic Acids from C. crenata Ethyl Acetate Extracts

By GC-MS and HPLC analyses, seven compounds were detected including gallic acid, protocatechuic acid, *p*-hydroxybenzoic acid, caffeic acid, ferulic acid, ellagic acid, and cinnamic acid (Table 7) (Supplementary Material, Figures S1 and S7) were identified and quantified, respectively. Caffeic acid and cinnamic acid were found as trace components (low amount <0.01 mg/g extract) (Table 7). In comparison with compound **1** (0.98 mg/g leaves), ellagic acid showed higher amount (2.26 mg/g leaves), whereas other phenolic acids had lower quantity (0.07–0.73 mg/g leaves) (Table 7).

## 3. Discussion

The present study investigated the inhibitory potential of *C. crenata* leaves for weed management. It was observed that the phytotoxicity varied among tested *L. sativa*, *R. sativus*, and *E. crus-galli*. The degree of inhibition was proportional to the applied doses (Table 1 and Table 3). The reduction on root growth might be due to allelochemicals’ suppression of mitosis [23]. Allelochemicals also resulted in irregular arrangement, organelle structures and finally damaged root cells [7]. In addition, growth of roots was directly affected by phytotoxins [24]. Basically, roots are less protected by a cuticle than shoots that could lead to higher accumulation of allelochemicals in root tissues, thus root growth is often inhibited in stronger levels than that of shoots [25].

The aqueous extracts of *C. crenata* adversely affected germination and seedling growth of *L. sativa*, *R. sativus*, and *E. crus-galli* (Table 1 and Table 2). *E. crus-galli* is known as one of the most problematic weeds in agricultural production, and frequently evolves herbicide resistance in fields [26]. In eastern China, rice yields can be reduced by as much as 10.8–25.3% by *E. crus-galli* at density > 6 plants/m^2^ in paddy fields [26]. Therefore, the biological control of this noxious weed is needed. In laboratory and greenhouse trials, the inhibition of *C. crenata* on the dicot weeds were found in stronger levels as compared to the monocot weeds. Thus, the inhibitory potential of *C. crenata* on weeds was selective and varied among weed species (Table 5 and Table 6).

Phenolic compounds are the most common allelochemicals found in plants [27]. The inhibitory potential of phenolic acids has been widely described in the literature [28]. Phenolic acids may increase the levels of active oxygen species by reducing antioxidant enzyme activity within cells [29], and inhibit the absorbance nutrients of plants in soil [7]. Chung et al. [11] screened 23 phenolic acids to evaluate their possibility to manage *E. crus-galli*, of which, *p*-hydroxybenzoic, ferulic, *p*-coumaric, and *m*-coumaric acids showed the highest suppressive activity on *E. crus-galli* emergence. Among them, *p*-hydroxybenzoic was reported to reduce the hydraulic conductivity and nutrient uptake of plant roots, resulting in growth inhibition [27]. Regosa et al. [30] examined the inhibitory effects of ferulic acid, gallic acid, *p*-coumaric, *p*-hydroxybenzoic acid, vanillic acid, and *p*-vanillin, and their mixture on emergence of several weeds. All of the phenolic acids showed reduction on germination and seedling growth of the selected weeds and the inhibitory levels varied among the tested compounds. However, the mixture of the phenolic acids showed negligible difference of inhibition as compared with individual phenolic acids. Inderjit et al. [31] investigated the joint action of *p*-hydroxybenzoic acid, *p*-coumaric acid, and ferulic acid on root growth suppression of *Lolium perennne* L. (perennial ryegrass). It was reported that the mixture of the three phenolic acids did not show stronger inhibition. Similar observations were observed on *p*-hydroxybenzoic acid, protocatechuic acid, vanillic acid, gentisic acid, gallic acid, caffeic acid, *p*-coumaric acid, syringic acid, ferulic acid and *o*-methoxybenzoic acid [32]. 

In this study, gallic acid, protocatechuic acid, *p*-hydroxybenzoic acid, caffeic acid, ferulic acid, ellagic acid, and cinnamic acid were found (Supplementary Material, Figures S1 and S7; Table 7). Of them, caffeic acid and cinnamic acid were in low quantities <0.01 mg, whereas other phenolic acids were in 0.30–3.24 mg/g extract or 0.07–2.26 mg/g leaves (Table 7). In our previous study, 13 phenolic acids and eight flavonoids were identified in different plant parts of *C. crenata* using 100% methanol for extraction, and they were suggested to correlate to the antioxidant properties of *C. crenata*. Of these, eight phenolic acids consisting of chlorogenic acid, *p*-hydroxybenzoic acid, vanillic acid, ferulic acid, sinapic acid, *p*-coumaric acid, benzoic acid, and ellagic acid were identified and quantified in the leaves [33]. However, in this study, an extraction solvent of 80% methanol was used because mixture of methanol and water yielded more water-soluble substances, and hence increased the inhibitory potential of the plant extracts [34,35]. The difference between extraction solvents may explain in the dissimilarity of phenolic acids and their contents, and flavonoids detected in GC-MS (Supplementary Material, Figure S7). 

These phenolic acids have been identified from many plants and their inhibitory potential were extensively investigated [11,27,28,29,30,31,32]. Except for the phenolic acids (Table 7), the isolation and purification of compounds other than phenolic acids, which were potent to inhibit weed growth, were also conducted. In this study, we did not re-examine the inhibitory activity of these known phenolic acids, but investigated the biological activities of the compound **1**. It was observed that 2α,3β,7β,23-tetrahydroxyurs-12-ene-28-oic acid showed stronger inhibition on shoot length and root length of *E. crus-galli* than *p*-hydroxybenzoic acid (Table 4). The suppression effect on root length of *E. crus-galli* was much stronger than that on *L. sativa* and *R sativus*, indicating that the inhibition of the compound **1** was selective for *E. crus-galli*. This isolated compound might be promising to develop natural herbicides to manage *E. crus-galli*. By column chromatography, an amount of 0.93 mg/g leaves of the compound **1** were purified. Ellagic acid had the maximum quantity (2.26 mg/g leaves), whereas other phenolic acids displayed much lower contents (0.07–0.73 mg/g leaves) (Supplementary Material, Figure S7). The chemical structure of the compound **1** was identified by analysis of ^1^H- and ^13^C-NMR (Supplementary Material, Figures S4–S6), IR (Supplementary Material, Figure S3), and LC-MS (Supplementary Material, Figure S2) data. In a previous study, proanthocyanidins, oligomeric flavonoids, were identified and quantified in high quantities (380 and 630 mg/g in water and ethanol extracts, respectively) in the inner skin of *C. crenata* [21]. However, proanthocyanidins were not found in this study, due to different extraction solvents and protocols that were used. 

This study reports for the first time the isolation of compound **1** from a plant. It was previously reported as a product from microbial transformation of asiatic acid by the fungus *Umbelopsis isabellina* but its biological activity has not been studied. Incubation of asiatic acid with *U. isabellina* provided two derivatives, including compound **1** and 2α,3β,7β,23-tetrahydroxyurs-11-ene-28,13-lactone [33]. Asiatic acid is a pentacylic triterpene acid, that mainly exists in *Centella asiatica* [36]. Many biological activities such as antitumor [36,37], neutroprotective [38], and anti-inflammatory effects [38] of this compound were reported. The C_2_ functional group of asiatic acid was modified and yielded 14 derivatives with enhanced hepatoprotective effects [39]. Pentacyclic triterpenes with many subgroups such as gammaceranes, hopanes, lupanes, oleananes, and ursane present a huge therapeutic potential [40]. HPLC is generally used for identification and quantification of known compounds by comparing the retention times and peak areas with those of the standards. In addition, the chemical structure of 2α,3β,7β,23-tetrahydroxyurs-11-ene-28,13-lactone was far different from the known phenolic acids detected in this study, and its spectra data was not available in the Wiley and NIST libraries. Therefore, the compound **1** could not be identified by HPLC and GC-MS. In this study, the constituent 2α,3β,7β,23-tetrahydroxyurs-11-ene-28,13-lactone was dissolved in methanol, showing that it was a soluble compound. Compound **1** was successfully isolated in this study perhaps due to the use of 80% methanol, that enhanced the yield of soluble substances [34,35]. Because GC-MS is broadly applied for volatile compounds, and the compound **1** was non-volatile, hence LC-MS was used to obtain its mass spectrum (Supplementary Material, Figure S2). Different extraction solvents should be tested to optimize the yield of 2α,3β,7β,23-tetrahydroxyurs-11-ene-28,13-lactone from *C. crenata* and to search for potent compounds other than the known phenolic acids, and proanthocyanidins [21], and compound **1** to explore potential uses of *C. crenata*.

## 4. Materials and Methods

### 4.1. General Experimental Procedures and Instrumentation

Methanol, ethanol, hexane, ethyl acetate, sulphuric acid, and hydrochloric acid were ordered from Merck Co., Ltd. (Tokyo, Japan). Fifteen analytical grade standard phenolic compounds including gallic acid, protocatechuic acid, catechol, chlorogenic acid, *p*-hydroxybenzoic acid, vanillic acid, caffeic acid, syringic acid, vanillin, ferulic acid, sinapic acid, *p*-coumaric acid, benzoic acid, ellagic acid, and cinnamic acid were purchased from Wako (Tokyo, Japan). 

Silica gel for column chromatography (60–100 mesh ASTM) and precoated TLC plates (layer thickness 0.25 mm), were purchased from Merck (Darmstadt, Germany). Visualization of the TLC spots was performed using 5% H_2_SO_4_ in ethanol spray reagent. Melting points were measured on an Electrochemical Engineering (Delhi, India) melting point apparatus. Optical rotation was evaluated on an Autopol model polarimeter (Rudolph, Hackettstown, NJ, USA). Ultraviolet-visible spectroscopy was measured with a TU-1800_PC_ UV-vis spectrophotometer (Shimadzu, Co., Ltd., Tokyo, Japan). Both ^1^H- and ^13^C-NMR spectra were recorded in CD_3_OD on a model 500 spectrometer (Bruker, Avance-500, Karlsruhe, Germany) operating at 500 and 125 MHz, respectively. Electrospray ionization (ESI) mass spectra were recorded in positive mode on a LC-MS (Thermo Scientific LTQ Orbitrap XL, Bremen, Germany) mass spectrometer using a standard ESI source coupled with LC separation system and HR-ESI MS (ESI-HRMS) in positive mode were recorded on an Agilent 6520 QTOF system (Agilent Technologies, Washington, DC, USA). Infrared spectra (4000–400 cm^−1^) were recorded on a Shimadzu 8201 PC FT-IR spectrophotometer (Shimadzu Co., Ltd., Tokyo, Japan). The evaporation of extracts was performed using a rotary evaporator (SB-350, EYELA, Tokyo, Japan).

### 4.2. Plant Materials

*C. crenata* leaves were collected from different trees in a campus affiliated with Hiroshima University (Higashi Hiroshima, Japan), in May 2016. The samples were well cleaned with tap water, and treated with 1% of NaClO in 5 min to remove the microbial, dried at 30 °C until the moisture was reduced to 12–14%. The dried leaves were ground by a miller into fine powder. Seeds of *Raphanus sativus* L. (radish) and *Lactuca sativa* L. (lettuce) were purchased from Taki Co. Ltd. (Kyoto, Japan). Seeds of *Echinochloa crus-galli* (L.) Beauv. (barnyardgrass) were collected from paddy fields in Hiroshima (Japan) in 2015. Empty seeds were removed by floatation in distilled water. The healthy seeds were dried and then kept at −20 °C for further experiments. Before use, seeds were sterilized with 0.1% sodium hypochlorite for 30 min and washed three times with distilled water. The germination tests of all seeds were conducted, the germination rate was >80%.

### 4.3. Extraction and Isolation of the Compound **1** by Column Chromatography

Dried *C. crenata* leaf powder (150 g) was immersed in methanol (1 L, 80%) for 48 h at 25 °C, and then the supernatant was concentrated to yield 35 g of extract. The methanol extract was subjected to normal phase column chromatography using silica gel (high purity grade Davisil Grade 635 pore size 60–100 Å mesh, 350 g, 4.0 cm × 80 cm, flow rate drop by drop) yielded 100 fractions (each fraction 200 mL) as follows: fractions (frs.) 1–5 in hexane, frs. 6–15 in hexane–EtOAc (9:1), frs. 16–25 in hexane: EtOAc (8:2), frs. 26–35 in hexane–EtOAc (7:3), frs. 36–45 in hexane–EtOAc (6:4), frs. 46–55 in hexane–EtOAc (5:5), frs. 56–65 in hexane–EtOAc (4:6), frs. 66–75 in hexane–EtOAc (3:7), frs. 76–85 in hexane–EtOAc (2:8), frs. 86–95 in hexane–EtOAc (1:9), frs. 96–100 in EtOAc. Fractions 46–55 were solidified and dissolved in chloroform, and filtered through a sintered funnel to yield pure compound **1** (140 mg, 0.93 mg/g leaves, Figure 1).

### 4.4. Spectral Data of Compound **1** Isolated from C. crenata Leaf

*2α*,*3β*,*7β*,*23-Tetrahydroxyurs-12-ene-28-oic acid* (**1**): White powder; m.p. 263–264 °C; $[\alpha {]}_{D}^{25}$ + 6.98 (*c*, 0.168, MeOH). IR (KBr) ν _max_ (cm^−1^): 3416, 2962, 1706, 1638, 1462,1379, 1260 (Supplementary Material, Figure S3); ^1^H-NMR (MeOD; 500 MHz): δ 2.28–2.31, 1.31–1.36 (m, H-1), 4.21 (d, *J* = 9.6, H-2), 4.23 (d, *J* = 9.6, H-3), 2.05–2.07 (m, H-5), 2.16–2.18, 1.81–1.83 (m, H-6), 4.42 (dd, *J* = 5.4, 10.8, H-7), 1.75–1.78 (m, H-9), 2.07–2.10 (m, H-11), 5.57 (br, s, H-12), 2.81–2.83, 2.19–2.21 (m, H-15), 2.07–2.09, 2.15–2.17 (m, H-16), 2.68 (d, *J* = 11.4, H-18), 1.52–1.54 (m, H-19), 1.00–1.02 (m, H-20), 1.42–1.44, 1.31–1.33 (m, H-21), 1.92–1.94 (m, H-22), 3.72, 4.25 (d, each, *J* = 10.2, *J* = 10.2, H-23), 1.00 (s, H-24), 1.13 (s, H-25), 1.36 (s, H-26), 1.35 (s, H-27), 0.98 (d, *J* = 6.6, H-29), 0.90 (d, *J* = 6.0, H-30) (Supplementary Material, Figures S5 and S6); ^13^C-NMR (MeOD; 125 MHz):δ 47.8 (C-1), 68.6 (C-2), 78.0 (C-3), 43.5 (C-4), 47.7 (C-5), 18.3 (C-6), 33.1 (C-7), 40.1 (C-8), 48.2 (C-9), 38.4 (C-10), 23.7 (C-11), 125.4 (C-12), 139.2 (C-13), 42.6 (C-14), 28.6 (C-15), 24.8 (C-16), 48.0 (C-17), 53.5 (C-18), 39.4 (C-19), 39.4 (C-20), 31.0 (C-21), 37.4 (C-22), 66.5 (C-23), 14.4 (C-24), 17.5 (C-25), 17.5 (C-26), 23.9 (C-27), 179.9 (C-28), 17.5 (C-29), 21.4 (C-30); HR-ESI-MS *m*/*z* 527.3341 [M + Na]^+^ observed (Calcd. for C_30_H_48_O_6_Na; *m*/*z* 527.3348) (Supplementary Material, Figure S2). Compound **1** was reported previously as a microbial transformation product of a fungus [22], and its data was compared with the spectra data of this study.

### 4.5. Inhibitory Activity of C. crenata Leaf Powder 

Ten seeds of each *R. sativus, L. sativa*, and *E. crus-galli* were sown in Petri dishes (9 cm in diameter) lined with double-filter papers and moistened with 6 mL of distilled water. Leaf powder of *C. crenata* with an amount of each 0.1, 0.25, and 0.5 g were placed evenly in the Petri dishes. The control was treated with distilled water in the same method. All treatments were put in a growth chamber (16 h light/night period, 25 °C) with a completely randomized design. The numbers of germinated seeds, shoot length, and root length were determined after 7 days. 

### 4.6. Inhibitory Activity of C. crenata Aqueous Extract

Different quantities (1, 5, 10 and 15 g) of the dried powder were soaked subsequently in 100 mL distilled water at room temperature for 24 h, following a method described in Mahmoud et al. [41]. The leaf extracts were centrifuged 8000 rpm for 5 min and then filtered through Advantec filter papers (Toyo Roshi Ltd., Tokyo, Japan) to remove the plant traces and yielded 1, 5, 10 and 15% dilutions. An aliquot of 6 mL of each aqueous extract was added to Petri dishes (9 cm in diameter) lined with two layers of filter papers. Control was applied with distilled water in a similar manner. Ten healthy seeds of each *L. sativa*, *R. sativus*, and *E. crus-galli* were sown in the Petri dishes with four replications. All treatments were transferred to a growth chamber (16 h of light/night, 25 °C) (Biotron NC system, Nippon Medical & Chemical Instrument, Co. Ltd., Osaka, Japan). Each Petri dish was supplemented with 2 mL distilled water in subsequent days. After 7 days, number of germinated seeds, radicle, and hypocotyl were recorded. 

### 4.7. Inhibitory Activity of C. crenata Ethyl Acetate Extract

Five grams of the dried leaf powder were immersed in 300 mL of 80% methanol for 24 h. The supernatant was filtered and evaporated by a rotary evaporator (SB-350-EYELA) at 35 °C. Afterward, crude extract was hydrolyzed by NaOH 4 M at 50 °C for 4 h. After wards, the solution was filtered and adjusted to pH 1.5 with HCl 37%, and then extracted with ethyl acetate five times. Subsequently, the ethyl acetate extracts were filtrated and evaporated. The precipitate was dissolved in distilled water to achieve 1000 and 2000 mg/L doses, whilst the controls were distilled water. The inhibitory effects of these dilutions on germination and growth of *R. sativus, L. sativa*, and *E. crus-galli* were examined. All treatments were organized in a completely randomized design and kept in a growth chamber set at 25 °C with 16 h light/night cycle, conducted in thrice and repeated twice. Each Petri dish was replenished with 2 mL distilled water in subsequent days. After 7 days, the number of germinated seeds, the length of shoots, and the length of roots were noted. In addition, similar ethyl acetate extracts were evaporated, dissolved in methanol and acetone for high-performance liquid chromatography (HPLC) and gas chromatography-mass spectrometry (GC-MS) analyses, respectively.

### 4.8. Inhibitory Activity of the Isolated Compound **1**

Ten seeds of each *R. sativus*, *L. sativa*, and *E. crus-galli* were placed on Petri dishes (9 cm in diameter) lined with two layers of filter paper and 6 mL of different dilutions (250, 500, and 1000 mg/L) of the isolated compound **1** (2α,3β,7β,23-tetrahydroxyurs-12-ene-28-oic acid) were added. Similar treatments with *p*-hydoxybenzoic acid were conducted to compare the efficacy with the isolated compound **1**. The IC_50_ value was expressed in milimole (mM), as the required amount to inhibit 50% shoot height and root length of each *L. sativa*, *R. sativus*, and *E. crus-galli*. All treatments were transferred to a growth chamber at 25 °C with 16 h of light/night cycle. The height of shoots and length of roots were measured after 7 days.

### 4.9. Inhibitory Effects of C. crenata on Growth of Weeds in Natural Soil in Laboratory and in Greenhouse

Natural soil collected from a field in the Experimental Station of Hiroshima University, Japan. The soil was well mixed then sieved carefully to remove trash. An amount of 100 g of soil was put in a Petri dish (9 cm in diameter) and saturated with distilled water. Then an amount of 0.1, 0.25, and 0.5 g of leaf powders were added in the Petri dishes, respectively. Controls received distilled water only. All treatments were arranged in a completely randomized design and placed in a growth chamber at 25 °C with 16 h light/night cycle. Distilled water was added into the Petri dishes every day. After 20 days, number of weed plants was recorded. The weed plants were dried by an oven at 50 °C for 2 days to determine the dry weight.

Similar natural soil as mentioned above was used, and transferred to a greenhouse (temperature 27–32 °C, humidity 70–80%, affiliated to Hiroshima University, Higashi Hiroshima, Japan), and placed until dried. Afterward, the soil was sieved carefully to remove trash. The soil was put in plastic pots (20 cm in diameter and 10 cm in height), and saturated with tap water. This experiment was conducted following a method described in Hong et al. [10] with three treatments consisting of T1 (spreading of powder at the 3rd day after watering), T2 (spreading of powder at the 3rd and 13th day after watering) and T3 (spreading of powder at the 3rd, 13th, and 23rd day after watering) with a similar dose (1 ton/ha) for all treatments. Controls were treated only with tap water. Thirty days after watering, weed species, number of plants and dry weight of emerged weeds were recorded.

### 4.10. HPLC and GC-MS Analyses 

The phenolic composition in the ethyl acetate extract was analyzed by a HPLC system, (JASCO, Tokyo, Japan) consisting of a PU-2089 Plus pump, LC-Net II/ADC controller, UV-2075 Plus detector, J-Pak Symphonia C18 column (5 μm, 4.6 × 250 mm, 110 Å)], detected at 254 nm. The mobile phases were performed with solvent A (methanol 100%) and solvent B (water with 0.1% acetic acid). In the first 5 min, the gradient elution process started with the mobile phase A increasing from 5% to 10%, for the next 45 min, increased from 10% to 90%, and the last 10 min was 100% A. The flow rate was 1 mL/min. An amount of 5 μL of the extracts (2 mg/L) was injected to the HPLC. Quantification of the phenolic acids were based on the curve established from the HPLC profiles of different dilutions (1, 10, 50, 100, and 250 mg/L) of the standards. The phenolic acids in the extracts were identified and quantified by comparing the retention times and peak areas of the authentic phenolic acids, respectively (Supplementary Material, Figure S1).

The ethyl acetate extract was silylated following the procedure of Proestos and Komaitis [42]. The silylated extract was injected to a GC-MS system (single quadrupole, ISQ Thermo, Austin, TX, USA). The Agilent DB5MS column used was 30 m in length, 0.25 mm in wide, and 0.25 µm in thickness (Agilent Technologies, J & W Scientific Products, Folsom, CA, USA). The GC oven temperature program was as follows: 50 °C hold for 6 min, raised at 10 °C/min to 250 °C, and hold 3 min. The injector was set at 250 °C and the detector at 280 °C. The flow rate of carrier gas (helium) was maintained at 1.0 mL/min. The mass range was scanned from 20–650 amu. Identification of compounds was obtained by comparing the retention times (Supplementary Material, Figure S7) and the spectral data obtained from the Wiley and NITS library.

### 4.11. Statistical Analysis

The laboratory and greenhouse experiments were carried out with four replications and repeated twice. Data were analyzed by using one-way ANOVA using the Minitab 16.0 software (Minitab Inc., State College, PA, USA), of which concentration was the fix factor. Significant differences between means were examined by using Tukey’s test (*p* < 0.05) and expressed as means ± standard errors (SE). The inhibitory effects in laboratory and greenhouse trials were expressed as the inhibition over control (%), and calculated as follows: (1 − treatment value/control value) × 100. The inhibitory potential of the isolated compound **1** and *p*-hydroxybenzoic acid was expressed in IC_50_ value, which calculated from the percentage of inhibition over control of shoot height and root length of *L. sativa*, *R. sativus,* and *E. crus-galli* by 250, 500, and 1000 mg/L concentrations. Thus, the IC_50_ values were the quantities in millimole (mM) need to inhibit 50% shoot height and root length of the tested plants. Accordingly, the lower value of the IC_50_ value exhibit stronger inhibitory potential.

## 5. Conclusions

Findings of this study showed that *C. crenata* had strong inhibitory potential, and suppresses germination and growth of shoots and roots of *L. sativa*, *R. sativus*, and *E. crus-galli*. Leaves of *C. crenata* appear to be a promising material to manage weeds, especially dicot weeds. This study reported for the first the detection of 2α,3β,7β,23-tetrahydroxyurs-12-ene-28-oic acid in a plant and its inhibitory activities. The medicinal and pharmaceutical properties of the compound **1** should be further investigated.

## Figures and Tables

**Table 1:** Effects of *C. crenata* dried leaf powder on germination and root and shoot growth of *L. sativa*, *R. sativus*, and *E. crus-galli.*

Weight (g/Petri Dish)	Germination (%)	Shoot Height (cm)	Root Length (cm)
*L. sativa*			
Control	85.0a ± 4.1(0.0)	1.0a ± 0.4 (0.0)	2.5a ± 1.2 (0.0)
0.10	73.3a ± 6.7(13.7)	1.1a ± 0.5 (−10.0)	0.7b ± 0.8 (72.0)
0.25	36.7ab ± 18.5 (56.8)	0.5b ± 0.5 (50.0)	0.1b ± 0.3 (96.0)
0.50	0.0b ± 0.0 (100)	0.0c ± 0.0 (100)	0.0b ± 0.0 (100)
*R. sativus*			
Control	96.7a ± 3.3 (0.0)	1.6a ± 3.9 (0.0)	7.4a ± 2.2 (0.0)
0.10	93.3a ± 3.3 (3.5)	2.0a ± 5.4 (−25.0)	1.1b ± 0.6 (85.1)
0.25	43.3b ± 18.5 (55.2)	0.5b ± 0.4 (68.8)	0.0c ± 0.0 (100)
0.50	23.3b ± 8.8 (75.9)	0.2b ± 0.4 (87.5)	0.0c ± 0.0 (100)
*E. crus-galli*			
Control	86.7a ± 6.6 (0.0)	3.8a ± 1.0 (0.0)	8.6a ± 2.7 (0.0)
0.10	86.7a ± 3.3 (0.0)	2.7b ± 0.9 (29.0)	0.9b ± 0.7 (89.5)
0.25	73.3a ± 12.0 (15.4)	1.4c ± 0.6 (63.1)	0.2b ± 0.2 (97.1)
0.50	70.0b ± 5.8 (19.2)	0.7c ± 0.4 (81.9)	0.04b ± 0.1 (100)

Values are means ± SE (standard errors); Values in parentheses indicate inhibition and minus values were promotion over controls; Means with similar letters in a column are not significantly different (*p* < 0.05).

**Table 2:** Effects of *C. crenata* aqueous extracts on germination and shoot and root growth of *L. sativa*, *R. sativus*, and *E. crus-galli.*

Concentration (g/100 mL)	Germination (%)	Shoot Height (cm)	Root Length (cm)
*L. sativa*			
Control	93.3a ± 6.7 (0.0)	1.7a ± 0.3 (0.0)	4.1a ± 0.8 (0.0)
1	96.7a ± 3.3 (−3.6)	1.9a ± 0.5 (−11.8)	2.2b ± 0.6 (46.3)
5	90.0ab ± 3.3 (0.6)	1.0b ± 0.2 (41.2)	1.0c ± 0.4 (75.6)
10	80.0ab ± 5.7 (14.3)	1.0b ± 0.1 (41.2)	0.5c ± 0.2 (87.8)
15	66.7b ± 5.7 (28.5)	1.0b ± 0.1 (41.2)	0.3c ± 0.2 (92.7)
*S. sativus*			
Control	93.3a ± 3.3 (0.0)	1.8a ± 0.5 (0.0)	7.2a ± 2.7 (0.0)
1	100a ± 0.0 (−7.2)	2.2a ± 0.5 (−22.0)	2.7b ± 1.1 (62.5)
5	90.0a ± 5.7 (3.6)	1.3b ± 0.4 (27.9)	0.6c ± 0.4 (91.7)
10	60.0b ± 0.0 (35.7)	1.1b ± 0.1 (39.0)	0.1c ± 0.1 (98.6)
15	60.0b ± 0.0 (35.7)	0.8b ± 0.3 (55.7)	0.2c ± 0.2 (97.2)
*E. crus-galli*			
Control	93.3a ± 3.3 (0.0)	3.5a ± 0.6 (0.0)	4.9a ± 0.9 (0.0)
1	93.3a ± 3.3 (0.0)	3.7b ± 0.3 (−5.8)	1.1b ± 0.5 (77.6)
5	86.7a ± 5.7 (7.1)	2.7c ± 0.3 (22.8)	0.2c ± 0.1 (95.9)
10	93.3a ± 3.3 (0.0)	2.0c ± 0.3 (42.8)	0.0c ± 0.0 (100)
15	93.3a ± 3.3 (0.0)	1.6c ± 0.2 (54.2)	0.0c ± 0.0 (100)

Values are means ± SE (standard errors); Values in the parentheses are the percentage of inhibition over controls. Minus values are percentage of promotion over controls; Means with similar letters in a column are not significantly different (*p* < 0.05).

**Table 3:** Effects of *C. crenata* ethyl acetate extract on germinations and growth of *R. sativus*, *L. sativa*, and *E. crus-galli.*

Concentration (mg/L)	Germination (%)	Shoot Length (cm)	Root Length (cm)
*L. sativa*			
Control	95.0a ± 3.3 (0.0)	1.1a ± 0.2 (0.0)	3.5a ± 0.9 (0.0)
1000	95.0a ± 3.3 (0.0)	0.6b ± 0.1 (43.1)	0.5b ± 0.2 (85.7)
2000	72.5b ± 8.7 (23.7)	0.5c ± 0.1 (52.6)	0.0b ± 0.1 (100)
*R. sativus*			
Control	97.5a ± 2.9 (0.0)	2.4a ± 0.3 (0.0)	7.3a ± 2.3 (0.0)
1000	92.5a ± 2.9 (5.1)	2.4a ± 0.2 (0.0)	8.8a ± 2.2 (−19.9)
2000	100a ± 0.0 (−2.6)	2.3a ± 0.3 (4.2)	6.3b ± 2.0 (12.4)
*E. crus-galli*			
Control	96.7a ± 3.3 (0.0)	3.1a ± 0.4 (0.0)	4.9a ± 0.7 (0.0)
1000	95.0a ± 3.3 (1.8)	2.9a ± 0.5 (6.9)	2.6b ± 0.9 (46.6)
2000	96.7ab ± 3.3 (0.0)	2.9a ± 0.3 (6.9)	0.1c ± 0.1 (98.0)

Values are means ± SE (standard errors); Values in the parentheses are the percentage of inhibition over controls. Minus values are percentage of promotion over controls; Means with similar letters in a column are not significantly different (*p* < 0.05).

**Table 4:** Inhibitory potential of 2α,3β,7β,23-tetrahydroxyurs-12-ene-28-oic acid on shoot height and root length of *L. sativ*a, *R. sativus,* and *E. crus-galli, compared with p*-hydroxybenzoic acid.

Compounds	IC_50_ in mM for Inhibition of Shoot Height			IC_50_ in mM for Inhibition of Root Length		
	*L. sativa*	*R. sativus*	*E. crus-galli*	*L. sativa*	*R. sativus*	*E. crus-galli*
The compound **1**	1.33a ± 0.02	-	2.62a ± 0.04	1.36a ± 0.02	8.52b ± 1.6	0.41a ± 0.02
*p*-Hydroxybenzoic acid	2.47b ± 0.03	-	15.33b ± 1.5	1.41b ± 0.01	5.27a ± 1.3	2.11b ± 0.04

Values are means ± SE (standard errors); Values in the columns are the quantities (mM) need to inhibit 50% shoot height and root length of *L. sativa, R. sativus*, and *E. crus-galli*. Values in a column with similar letters are not significantly different (*p* < 0.05). -: measurements were not conducted.

**Table 5:** Effects of *C. crenata* dried leaf powder on plant number and dry weight of weeds in the natural soil in laboratorial trials.

Concentration (g)	Weed Number (Plants)			Dry Weight (mg)		
	Monocots	Dicots	Total	Monocots	Dicots	Total
Control	9.0a ± 0.6 (0.0)	19.7a ± 6.6 (0.0)	28.7a ± 0.3 (0.0)	7.8b ± 0.5 (0.0)	5.0a ± 0.5 (0.0)	12.8b ± 0.8 (0.0)
0.10	7.3ab ± 0.9 (18.9)	20.7a ± 2.7 (−5.1)	28a ± 2.5 (2.4)	7.9b ± 0.1 (−0.9)	5.3a ± 0.6 (−7.4)	13.2b ± 1.3 (−3.2)
0.25	4.3b ± 0.9 (52.2)	8.3b ± 0.9 (57.9)	12.7b ± 1.8 (55.7)	3.4c ± 0.3 (56.4)	2.1b ± 0.2 (57.7)	5.5c ± 0.2 (57.0)
0.50	5.7b ± 0.3 (36.7)	6.3b ± 1.3 (68.0)	12b ± 1.5 (58.2)	17.6a ± 0.8 (−125.6)	2.4b ± 0.3 (51.0)	20.0a ± 0.9 (−56.3)

Values are means ± SE (standard errors); Monocot weeds: *Cyperus microiria* Steud, *Fimbristylis littoralis* Gaudich, *Digitaria violascens* Link; Dicot weeds: *Eclipta prostrata* L., *Stellaria media* (L.) Vill., *Cardamine flexuosa* With; Values in the parentheses are percentage of inhibition over control; Minus values are percentage of promotion over control; Means with similar letters in a column are not significantly different (*p* < 0.05).

**Table 6:** Effects of *C. crenata* dried leaf powder on the emergence of weeds in the natural soil in a greenhouse.

Weed Species	Weed Number (Plants)				Dry Weight (mg)			
	Control	T1	T2	T3	Control	T1	T2	T3
Monocots								
*Cyperus microiria*	120.5a (0.0)	107.3a (11.0)	130.5a (−8.3)	135.8a (−12.7)	268.7b (0.0)	448.3a (−66.8)	426.9a −58.9)	333.0ab (−23.8)
*Fimbristylis littoralis*	23.0a (0.0)	23.3b (−1.1)	13.0a (43.5)	14.5a (37.0)	26.3a (0.0)	25.8a (1.8)	33.3a (−26.8)	31.0a (−18.5)
*Digitaria violascens*	5.5b (0.0)	12.3a (−123.6)	12.8a (−132.7)	11.3a (−105.5)	87.0c (0.0)	221.4a (−154.4)	183.9ab (−111.3)	115.0bc (−32.3)
Dicots								
*Eclipta alba*	5.8a (0.0)	2.0b (63.6)	2.0b (63.6)	0.3b (94.8)	14.0a (0.0)	6.9b (50.8)	5.7b (59.6)	4.7b (66.5)
*Stellaria media*	93.5a (0.0)	38.8c (58.5)	65.0b (28.5)	68.8b (26.4)	55.1b (0.0)	35.9b (34.8)	72.7a (−31.9)	76.5ab (−38.8)
*Cardamin flexuosa*	2.3a (0.0)	4.0a (−73.9)	0.0c (100)	0.0c (100)	5.5b (0.0)	20.0a (−190.9)	0.0c (100)	0.0c (100)

Values in the parentheses are percentage of inhibition over control; Minus values are percentage of promotion over control; Means with similar letters in a row are not significantly different (*p* < 0.05); T1 (spreading of powder at the 3rd day after watering); T2 (spreading of powder at the 3rd and 13th day after watering), and T3 (spreading of powder at the 3rd, 13th, and 23rd day after watering).

**Table 7:** Phenolic compound contents (mg/g) of the ethyl acetate extract of *C. crenata* leaf identified and quantified by GC-MS and HPLC, respectively.

Compounds	Retention Time (min)	Quantity (mg/g Extract)	Quantity (mg/g Leaves)
Gallic acid	12.6	3.15 ± 0.32a	0.71 ± 0.0.13b
Protocatechuic acid	17.05	0.49 ± 0.02c	0.11 ± 0.01c
*p*-Hydroxybenzoic acid	20.58	0.30 ± 0.01c	0.07 ± 0.00c
Caffeic acid	21.6	Trace; <0.01 mg	Trace; <0.01 mg
Ferulic acid	24.58	3.24 ± 0.31a	0.73 ± 0.10b
Ellagic acid	28.25	1.74 ± 0.03b	2.26 ± 0.21a
Cinnamic acid	29.62	Trace; <0.01 mg	Trace; <0.01 mg

Each value represents the mean of five replicates ± SE (standard errors). Means with similar letters in a column are not significantly different at *p* < 0.05; tr: trace.

## Supplementary Materials

The following are available online.
