# Supplementary material for: Weed Suppressing Potential and Isolation of Potent Plant Growth Inhibitors from Castanea crenata Sieb. et Zucc
Source: Molecules. 2018 Feb 7;23(2):345. doi: 10.3390/molecules23020345 (PMC6017574; doi:10.3390/molecules23020345)
Supplement: Supplementary file 1 [file molecules-23-00345-s001.pdf]

Weed Suppressing Potential and Isolation of Potent Plant Growth Inhibitors from *Castanea crenata* Sieb. et Zucc

Phung Thi Tuyen [1,2], Tran Dang Xuan [2,*], Truong Thi Tu Anh[2], Truong Mai Van [2], Ateeque Ahmad [3], Abdelnaser Abdelghany Elzaawely [4], andTran Dang Khanh [5],

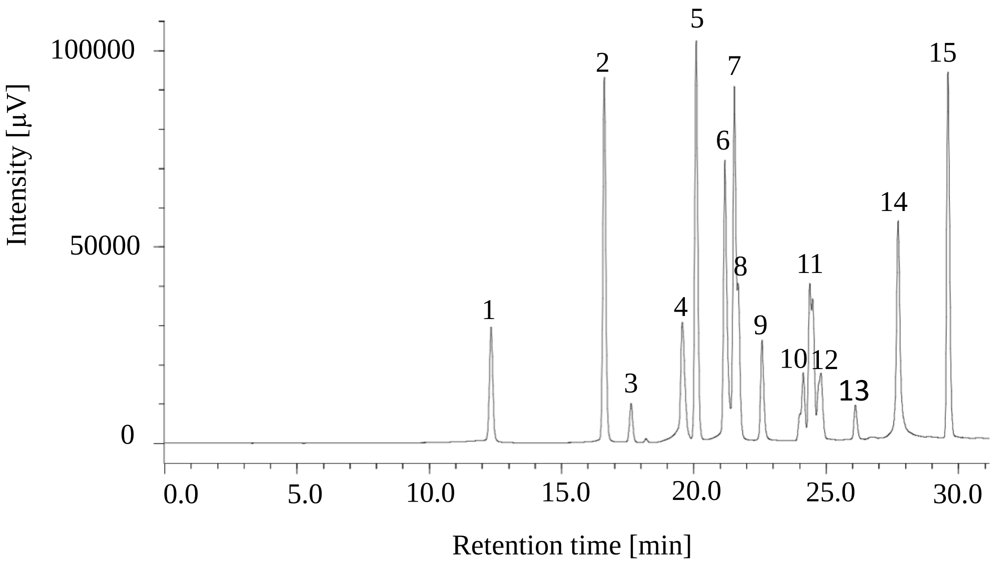

(a)

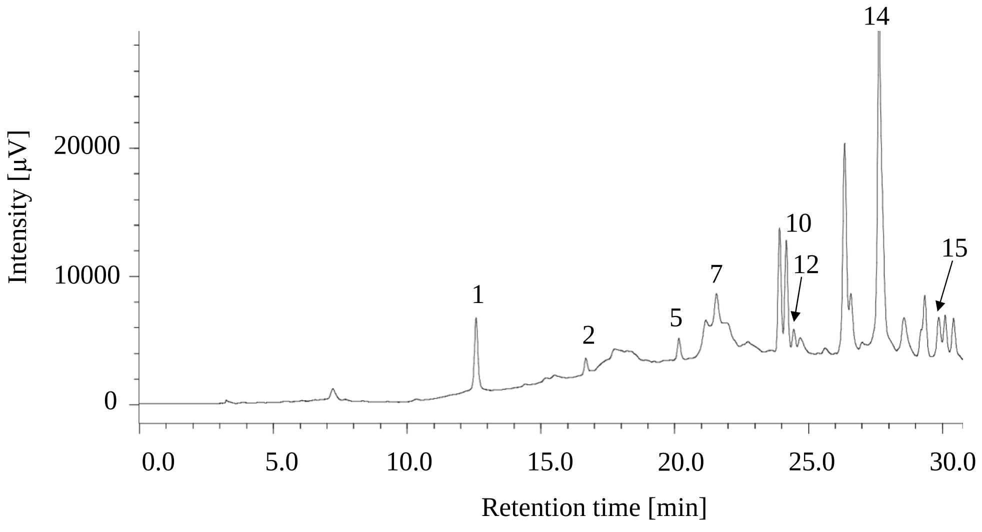

(b)

Supplement Material, Figure S1. Chromatograms of phenolic standard mixture (a) and ethyl acetate extract (b). Peaks 1: gallic acid; 2: protocatechuic acid; 3: catechol; 4: chlorogenic acid; 5: *p*-hydroxybenzoic acid; 6: vanillic acid; 7: caffeic acid; 8: syringic acid; 9: vanillin; 10: ferulic acid; 11: sinapic acid; 12: *p*-coumaric acid; 13: benzoic acid; 14: ellagic acid; 15: cinnamic acid.

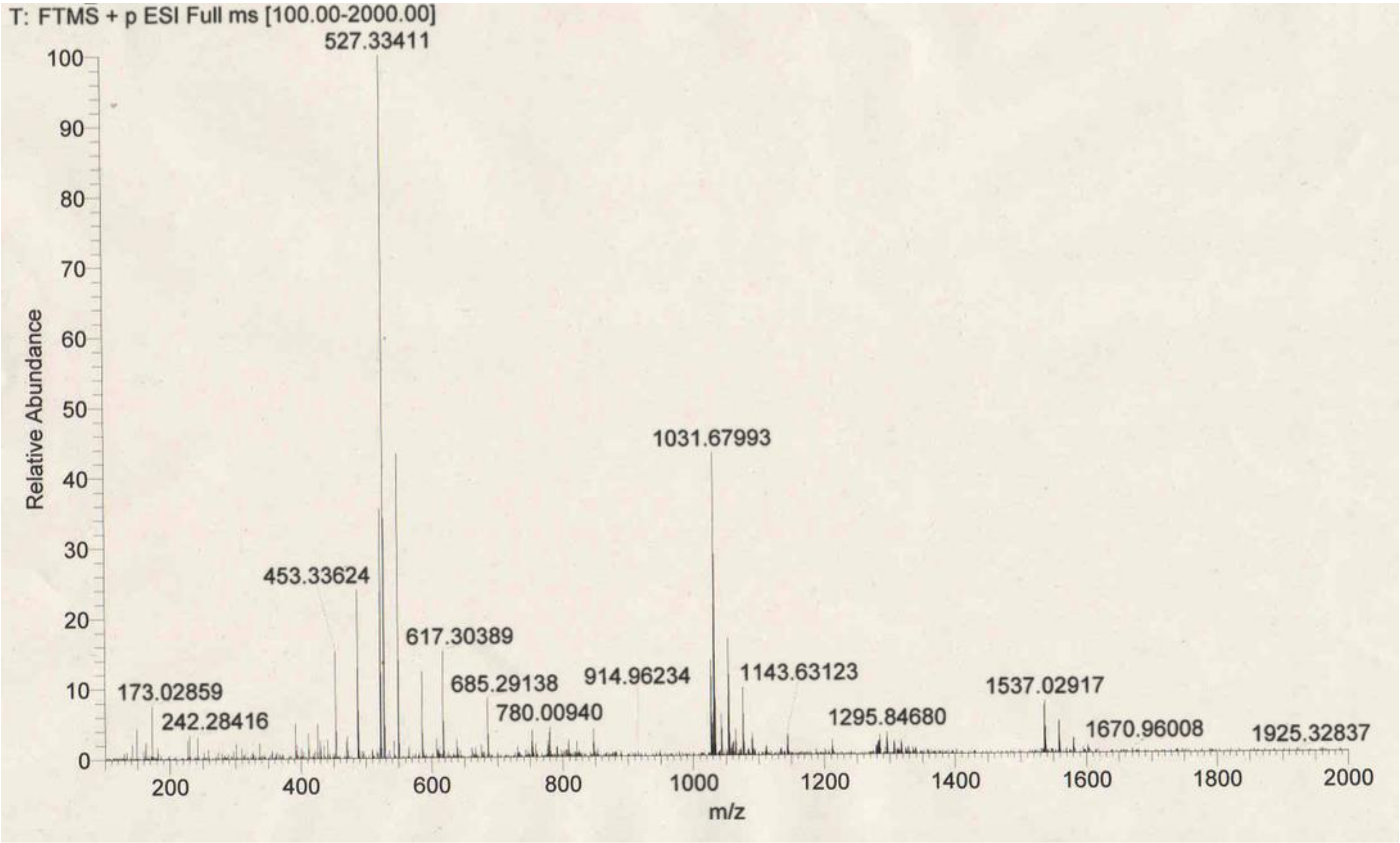

Supplement Material, Figure S2. LC-MS spectrum of 2a,3b,7b,23-tetrahydroxyurs-12-ene-28-oic acid (compound **1**) in positive mode

AA-T2

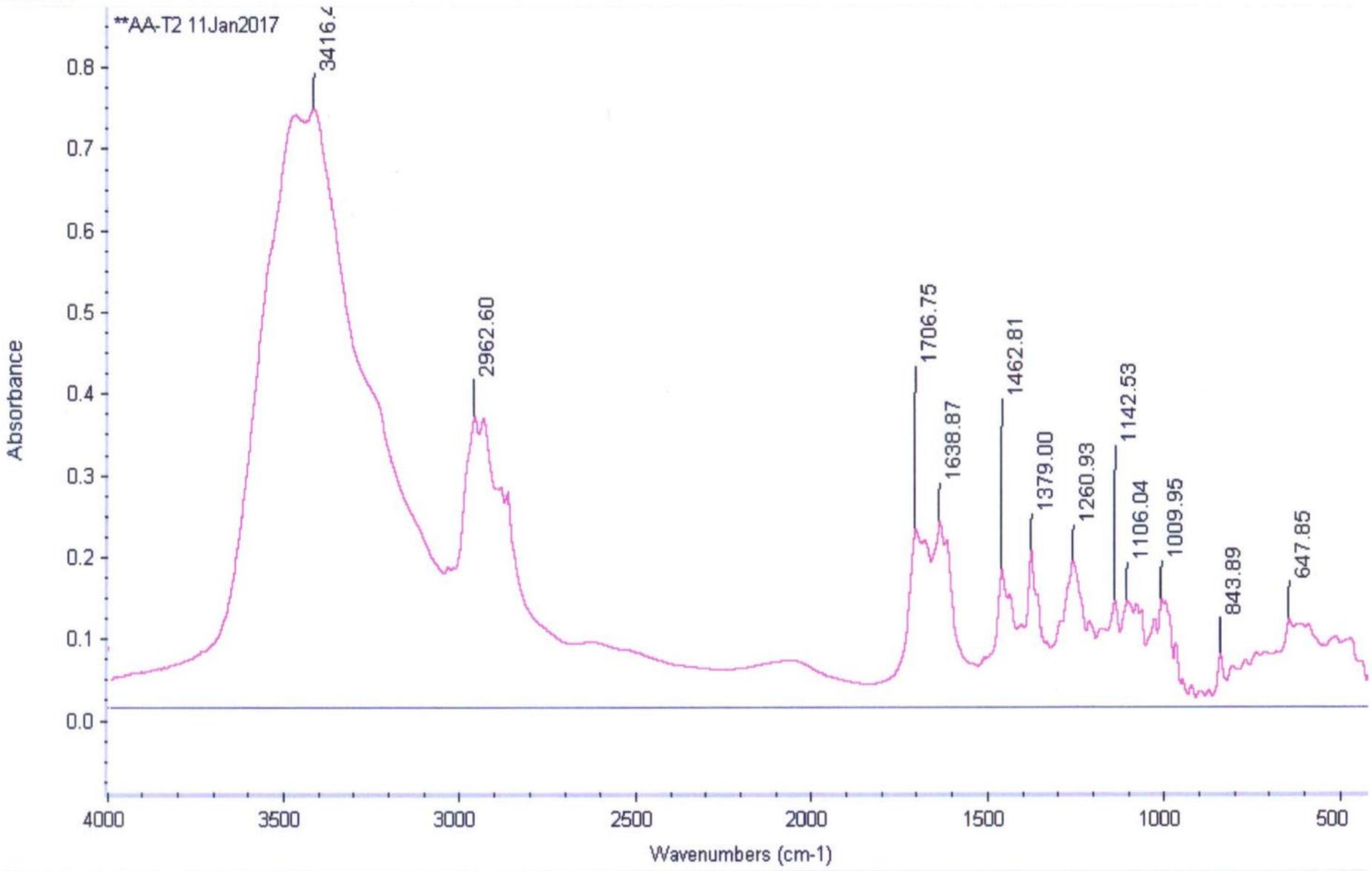

Figure AP2.

Supplement Material, Figure S3. Infrared spectrum (IR) of 2a,3b,7b,23-tetrahydroxyurs-12-ene-28-oic acid (compound **1**).

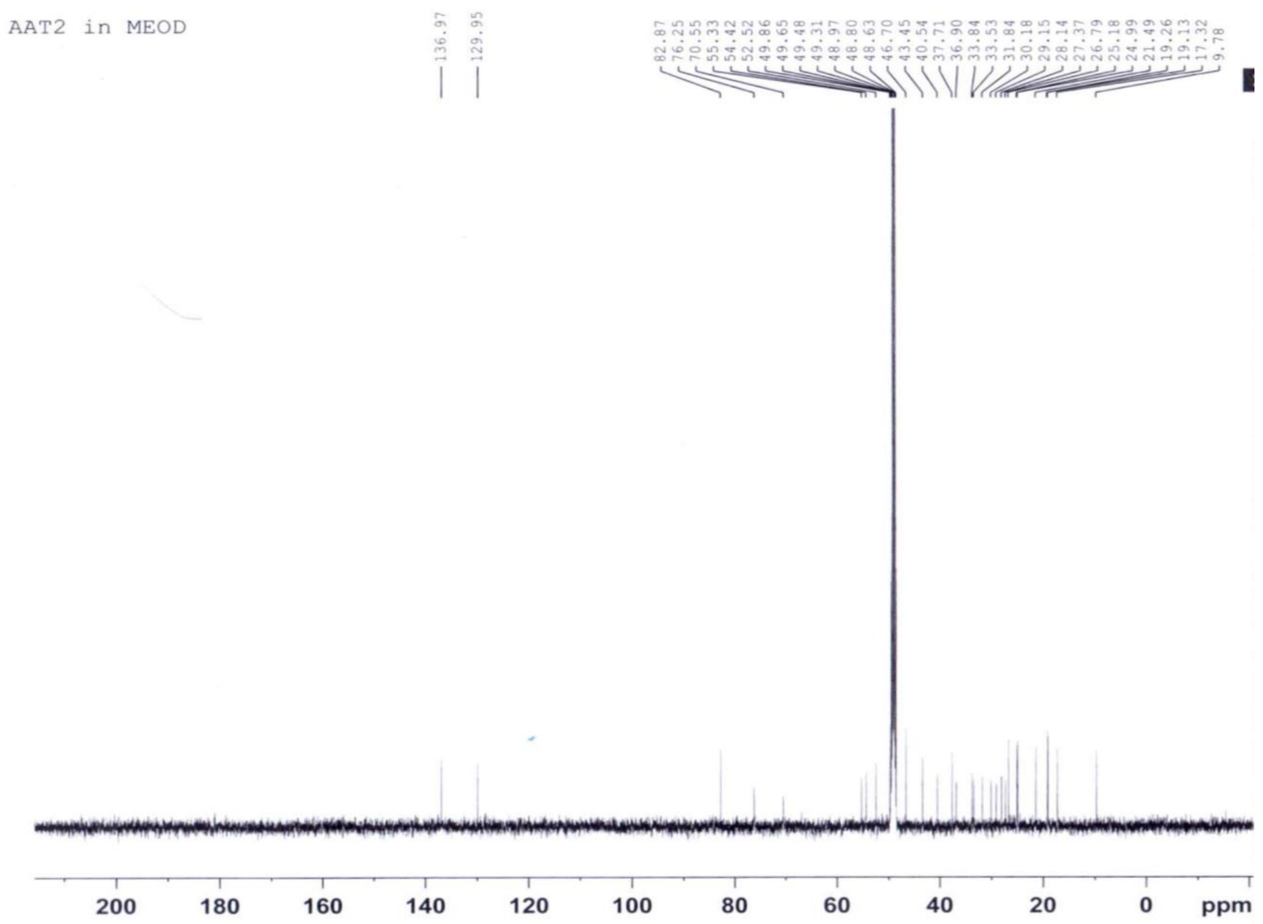

Supplement Material, Figure S4. ^{13}C NMR spectrum of 2a,3b,7b,23-tetrahydroxyurs-12-ene-28-oic acid (compound **1**) in CD_3OD

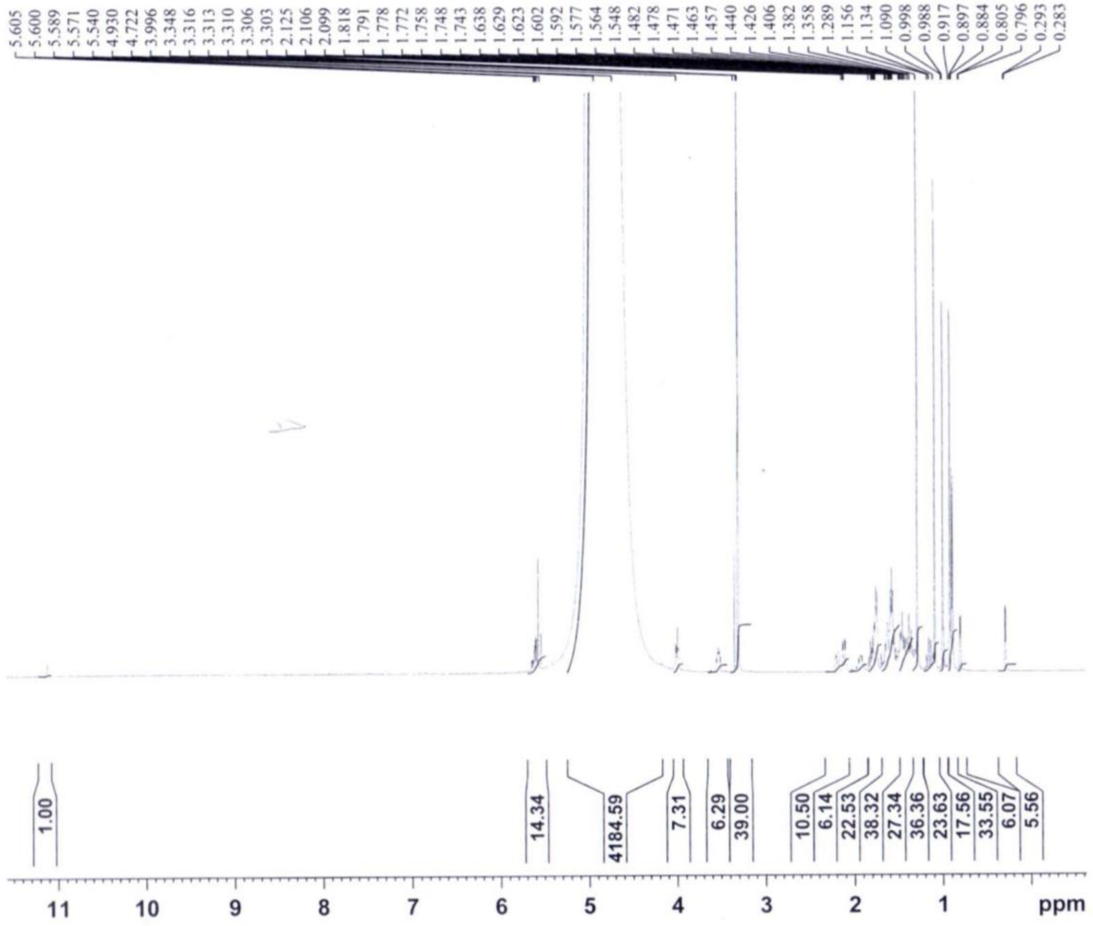

Supplement Material, Figure S5. ^{1}H NMR spectrum of 2a,3b,7b,23-tetrahydroxyurs-12-ene-28-oic acid (compound **1**) from 1–10 ppm

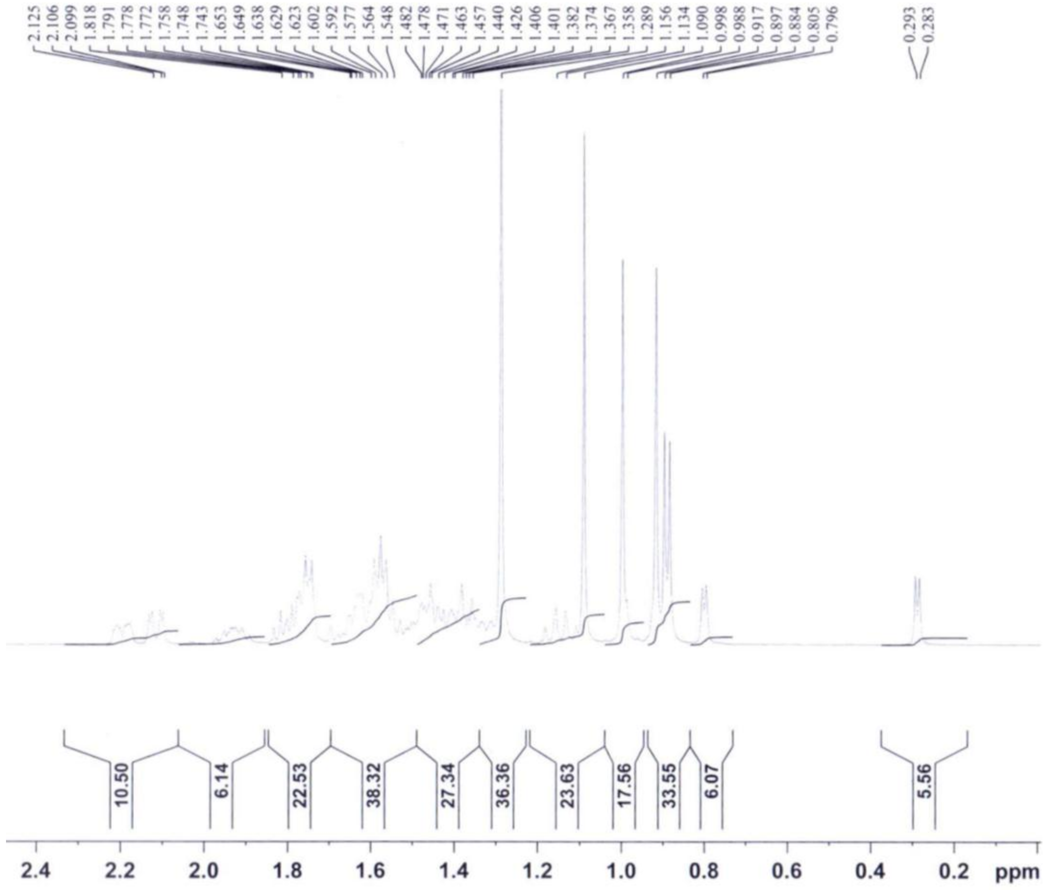

Supplement Material, Figure S6. ^{1}H NMR spectrum of (2a,3b,7b,23-tetrahydroxyurs-12-ene-28-oic acid (compound **1**) expansion of the signal from 0.2–2.4 ppm in CD_3OD

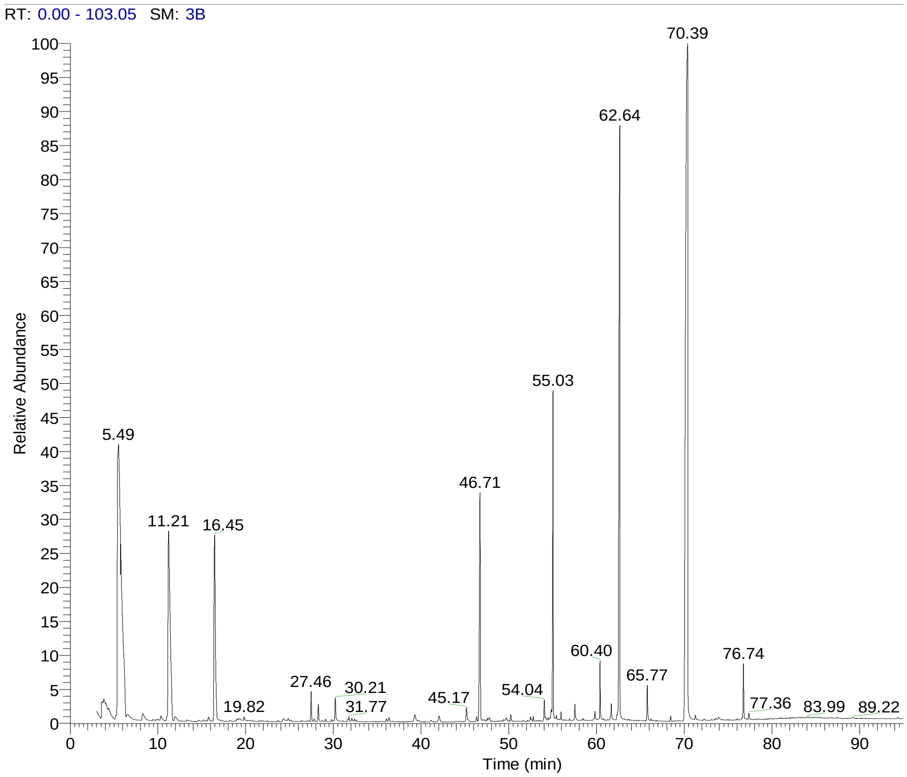

(a)

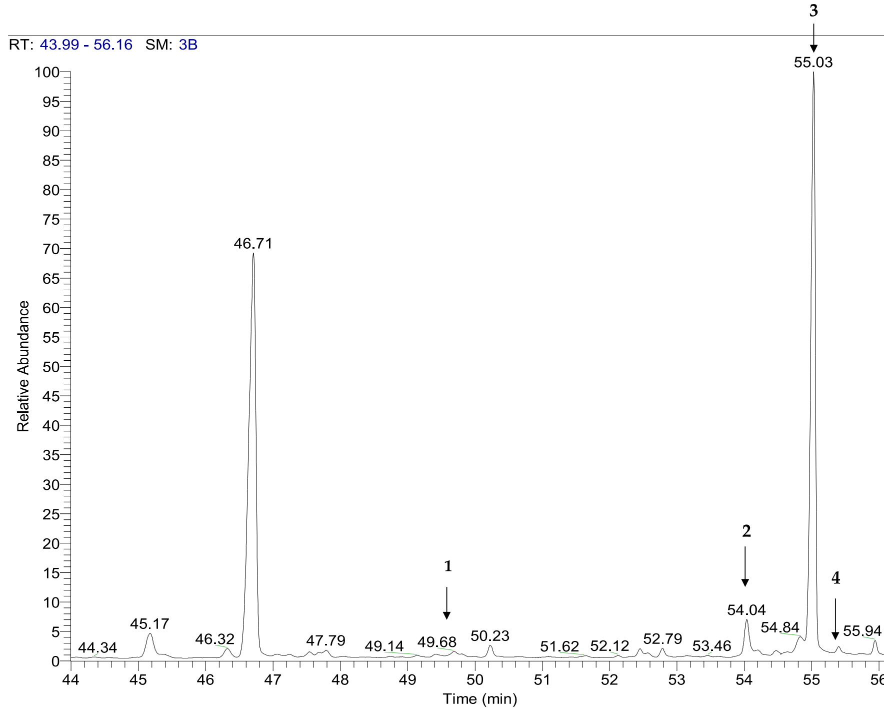

(b)

Supplement Material, Figure S7. GC-MS Chromatogram of *C. crenata* ethyl acetate leaf extract (a) from 0 to 90 (min), and (b) from 44 to 56 (min). Peak **1**: Protocatechuic acid; **2**: *p*-hydrobenzoic acid; **3**: Gallic acid; **4**: Caffeic acid.
